# Childbed Mortality

**Published:** 1915-12-04

**Authors:** 


### Childbed Mortality.

According to Sir Halliday Croom, three-fourths of the deaths following labour are due to septic poisoning—a cause entirely preventable. While formerly the death-rate from this cause in maternity hospitals was from 15 to 25 per cent., the influence of Lister's teaching reduced it to nil. In private practice the position is even yet very far from satisfactory, as antiseptic precautions cannot be enforced on those attending labours; and again, these precautions are not always easy to procure in private houses. Great benefit had followed the Midwives Act, the mortality from puerperal sepsis in England having fallen to 1.64 per thousand, whereas in Scotland, where the Midwives Act does not apply, no such favourable change has taken place. He sympathised with the efforts which are now being made to extend the operation of the Act to Scotland. Further, he claimed that the figures showed an incontrovertible claim for the maternity hospital. But these hospitals needed support, and Sir Halliday spoke in favour of the proposal made to the Insurance Commissioners to utilise them in association with the approved societies. Unless some such plan was adopted the hospitals would have to be closed, and this would involve serious prejudice to the teaching of obstetrics.—*The Medical Magazine.*

